# Predictive roles of inflammatory indicators (NLR, PLR, LMR and SII) in poststroke depression rehabilitation: a systematic review

**DOI:** 10.3389/fneur.2026.1890435

**Published:** 2026-10-05

**Authors:** Hao Dai, Ming Cheng

**Affiliations:** Department of Rehabilitation Medicine and Pain Management, Chengdu Jinniu District People's Hospital, Chengdu, Sichuan Province, China

**Keywords:** depression, diagnosis, inflammation, inflammatory indicators, ischemic stroke

## Abstract

Acute ischemic stroke (AIS) is a leading cause of mortality and long-term disability worldwide. Almost all AIS survivors suffer physical disabilities and neuropsychiatric disturbances, among which poststroke depression (PSD) is a typical poststroke complication. The development of new tools to diagnose PSD at the early stage and predict its long-term outcome has attracted the attention of clinicians and researchers. Neuroinflammation has been increasingly considered a critical modulator of stroke evolution. Following an IS, an inflammatory response arises in the CNS, characterized by the activation of microglia; the recruitment of peripheral immune cells, including neutrophils, monocytes, and lymphocytes; and the release of proinflammatory cytokines. In this review, we present a comprehensive and critical examination of the diagnostic and predictive roles of inflammatory indicators, including the neutrophil-to-lymphocyte ratio (NLR), platelet-to-lymphocyte ratio (PLR), lymphocyte-to-monocyte ratio (LMR), and systemic immune-inflammation index (SII), in PSD. A systematic literature search on PubMed, Google Scholar, Web of Science, and CNKI was performed up to January 31, 2026. Quality assessment was conducted using the Newcastle–Ottawa Scale (NOS), and heterogeneity was evaluated using the *I*^2^ statistic. Nine original studies involving 2,626 patients were included. Compared with non-PSD (NPSD) patients, PSD patients had a significantly greater NLR (*MD* = 0.47, 95% *CI* [0.39, 0.56], *I*^2^ = 49%), PLR (*MD* = 20.79, 95% *CI* [12.25, 29.33], *I*^2^ = 90%), and SII (*MD* = 123.92, 95% *CI* [68.85, 178.98], *I*^2^ = 70%) and a significantly lower LMR (*MD* = −0.21, 95% *CI* [−0.34, −0.08], *I*^2^ = 64%). Most included studies were rated as moderate-to-high quality on the basis of their NOS scores. These findings suggest that the NLR, PLR, LMR, and SII may serve as accessible peripheral blood biomarkers for early risk assessment of PSD, although further research is needed to establish optimal cutoff values and validate their predictive utility across different stroke subtypes.

## 1 Introduction

The World Stroke Organization's 2025 Global Stroke Fact Sheet revealed that from 1990 to 2021, the global burden of stroke significantly increased. In terms of absolute case numbers, stroke incidence increased by 70.0%, mortality by 44.0%, prevalence by 86.0%, and disability-adjusted life years (DALYs) by 32.0%. The majority of the global stroke burden (87.0% of deaths and 89.0% of DALYs) is concentrated in low-income and lower-middle-income countries (1). Poststroke complications are common, including (1) poststroke depression (PSD), with an overall incidence of 27% (2); (2) poststroke cognitive impairment, with an overall incidence of 39%-47% (3); and (3) poststroke dysphagia, with an overall incidence of 46.6% (4). Patients with PSD often have reduced rehabilitation compliance and quality of life (5), with a mortality rate as high as 50% (6). Current clinical diagnosis of PSD relies primarily on structured interviews combined with the use of various depression rating scales (7).

Existing studies have explored the use of biomarkers to predict PSD. For example, Liang et al. (8) reported that elevated homocysteine levels may be an independent predictor of depression in patients during the early phase of acute ischemic stroke (AIS). However, current diagnostic methods still have notable limitations: most involve subjective patient input and struggle to differentiate PSD from similar complications, such as post-stroke pseudobulbar affect. Additionally, as stroke is categorized into ischemic and hemorrhagic subtypes, research on the associations between different stroke subtypes and PSD is limited (9). In summary, exploring specific and effective early predictors for PSD would enable precise identification and early intervention for this complication.

Increasing evidence confirms that inflammation plays a dual role in the progression of AIS and the pathological mechanisms of secondary damage. On the one hand, inflammation can expand the infarct area, disrupt the integrity of the blood–brain barrier (BBB), promote leukocyte and protein extravasation, and induce neuronal and glial cell death through the release of proinflammatory factors, reactive oxygen species (ROS), and excitotoxic substances. On the other hand, inflammation can enhance the tolerance of the brain to ischemic injury by mediating tissue repair (including the clearance of necrotic tissue, angiogenesis, and neural repair) (10). Monitoring poststroke inflammatory levels is considered a potential strategy for assessing patient prognosis and complications. For example, Zhou et al. (11) analyzed 26 studies involving 6,573 patients with acute stroke and reported that serum levels of IL-1β, IL-6, IL-18, TNF-α, and IFN-γ were significantly greater in PSD patients than in non-PSD (NPSD) patients. Notably, IL-6 levels remained associated with PSD diagnosis at 1, 3, and 6 months post-stroke, suggesting that inflammatory cytokines could serve as biomarkers for PSD. C-reactive protein (CRP), an evolutionarily conserved plasma protein, is present at significantly elevated concentrations during inflammation, making it a classic acute-phase inflammatory marker (12). Chen et al. (13) studied 3,294 patients with ischemic stroke (most of whom had AIS). They reported that CRP concentrations were significantly greater in AIS patients than in healthy individuals. Additionally, CRP levels in PSD patients at the early stage were notably higher than those in NPSD patients, indicating that CRP levels hold diagnostic and prognostic significance for PSD.

Inflammatory parameters, including the neutrophil-to-lymphocyte ratio (NLR), platelet-to-lymphocyte ratio (PLR), lymphocyte-to-monocyte ratio (LMR), and systemic immune-inflammation index (SII), have been identified as independent clinical predictors and are widely used in the diagnosis and severity assessment of inflammatory diseases (14). For example, Xin et al. (15) reported that the NLR can be used for the early identification of sepsis, distinguishing sepsis from non-infectious systemic inflammatory response syndrome, and assessing disease severity. Erdogan et al. (16) reported that the SII is an independent predictor of functionally significant coronary artery stenosis in patients with chronic coronary heart disease and can significantly predict hemodynamically severe obstruction. Karauzum et al. (17) reported that the LMR can predict postoperative renal injury in patients with acute coronary syndrome; Zhang et al. (18) reported that the PLR and NLR can serve as prognostic biomarkers for elderly patients with chronic obstructive pulmonary disease and respiratory failure. Moreover, studies have revealed that inflammatory parameters, including the NLR, PLR, LMR, and SII, are closely associated with stroke severity and prognosis. For instance, Zhang et al. (19) revealed that elevated NLR and PLR, along with reduced LMR, are independent risk factors for poor functional outcomes at discharge in stroke patients, whereas the NLR and LMR are correlated with the length of hospital stay. Huang et al. (20) confirmed that high SII and SIRI indices are associated with an increased risk of stroke severity and that the SII helps predict adverse clinical outcomes of stroke.

PSD is a common complication that affects the long-term recovery of stroke patients, and its pathogenesis is related to inflammatory disorders in the body (21). However, a systematic review of the predictive value of the NLR, PLR, LMR, and SII in PSD is lacking. This review summarizes existing research on the predictive value of four inflammatory indicators in PSD, compares the predictive efficacy of each indicator, analyzes the factors influencing their efficacy, proposes directions for clinical translation and research gaps, and provides references for subsequent mechanistic studies and clinical applications.

## 2 Literature search and analysis

### 2.1 Literature search strategy

We conducted searches using the PubMed, Google Scholar, Web of Science, and CNKI databases. We combined subject headings and free terms for literature retrieval. The main search terms included “neutrophil-to-lymphocyte ratio”, “platelet-to-lymphocyte ratio”, “lymphocyte-to-monocyte ratio”, “systemic immune-inflammation index”, and “poststroke depression”. The abbreviations of these terms, “NLR”, “PLR”, “LMR”, “SII”, and “PSD”, were also used as search terms. The search period spanned from the establishment of the databases to January 31, 2026.

### 2.2 PRISMA guidelines

This systematic review was conducted and reported in accordance with the Preferred Reporting Items for Systematic Reviews and Meta-Analyses (PRISMA) 2020 statement (Figure 1). The detailed search strategies for each database are provided in Supplementary Table S1. The PRISMA 2020 main checklist (27 items) is provided in Supplementary Table S2, and the PRISMA 2020 for Abstracts checklist (12 items) is provided in Supplementary Table S3. This review was not registered in PROSPERO or any other public registry.

**Figure 1:**
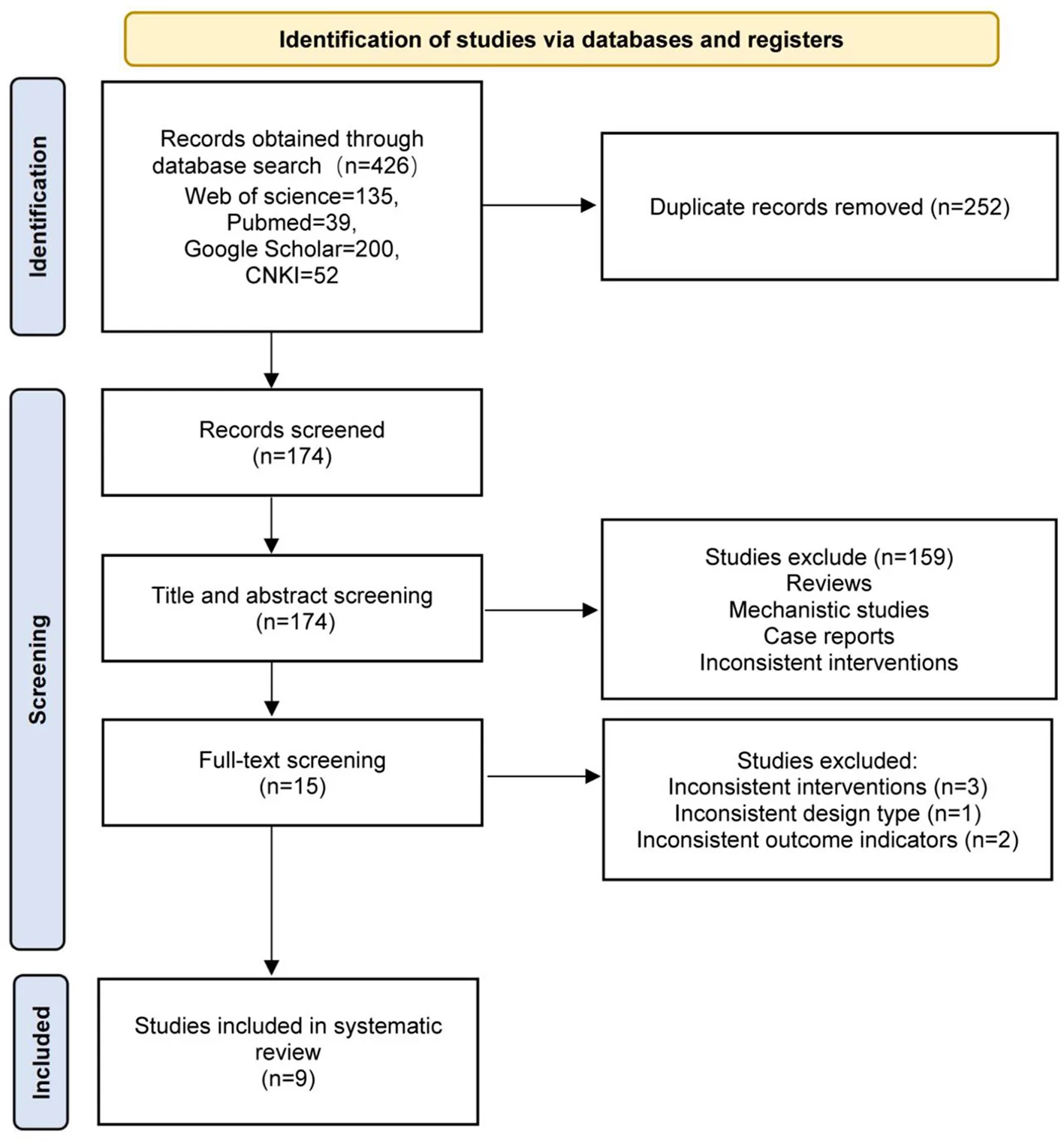
PRISMA 2020 flow diagram of the study selection process.

### 2.3 Inclusion and exclusion criteria

In accordance with the criteria of the PICOS framework, eligible literature was screened to ensure systematic retrieval. Prospective and retrospective cohort studies evaluating the prognostic role of the NLR, PLR, LMR, SIRI, and SII in the development of PSD were included. The samples of the included articles were examined for sufficient informativeness, including the number of patients in the PSD group and the NPSD group, as well as the mean difference (MD) in the NLR, PLR, LMR, SIRI, and SII in both groups. Studies reporting medians and interquartile ranges or/and ranges were also included, and statistical methods were used to estimate the MD. Animal and case–control studies, letters to editors, case series, case reports, conference papers, studies with overlapping data, and duplicate studies were excluded. Study selection and data extraction were independently performed by two reviewers, with disagreements resolved through discussion or consultation with a third reviewer. No automation tools were used in either process.

### 2.4 Quality assessment

The methodological quality of the included cohort studies was independently assessed by two reviewers using the Newcastle–Ottawa Scale (NOS), which evaluates studies across three domains (selection, comparability, and outcome) with a maximum total score of 9 points. Studies with scores ≥7, 4–6, and ≤ 3 were considered high, moderate, and low quality, respectively. Any disagreements were resolved through discussion or by consulting a third reviewer. The detailed NOS scores are presented in Table 1. Publication bias was not formally assessed because of the limited number of studies (< 10) per analysis, as funnel plots and Egger's test are unreliable with fewer than 10 studies. Formal GRADE assessment was not performed, as this review was exploratory in nature and aimed to summarize the available evidence rather than to provide clinical recommendations.

**Table 1:** Assessment of the quality of the included cohort studies using the Newcastle–Ottawa Scale (NOS).

References	Selection			Comparability			Outcome		Total	Quality
	Representativeness of the exposed cohort	Selection of the non-exposed cohort	Ascertainment of exposure	Outcome not present at start	Comparability of cohorts	Assessment of outcome	Follow-up long enough	Adequacy of follow-up	Score (/9)	
Chen et al. (25)	1	1	1	1	2	1	1	1	9	High
Chen et al. (30)	1	1	1	1	2	1	1	1	9	High
Ding et al. (32)	1	1	1	1	2	1	1	1	9	High
Han et al. (28)	1	1	1	1	1	1	1	1	8	High
Hu et al. (27)	1	1	1	1	1	1	1	1	8	High
Hu et al. (29)	1	1	1	1	2	1	1	1	9	High
Huang et al. (26)	1	1	1	1	2	1	1	1	9	High
Su et al. (24)	1	1	1	1	2	1	1	1	9	High
Xiao et al. (31)	1	1	1	1	1	1	1	1	8	High

### 2.5 Statistical analysis

The analysis was performed using Review Manager 5.3 statistical software. MD with a 95% confidence interval (CI) was used as the effect measure for all continuous outcomes, as all included studies measured the NLR, PLR, LMR, and SII using identical units. The combined SMDs measured by the *Z*-test with *P* < 0.05 were considered to indicate statistical significance. Heterogeneity across studies was measured on the basis of the *P*-value of the chi-square *I*^2^ statistic. If *P* was < 0.10 or *I*^2^ was >50%, high heterogeneity was indicated, and a random-effects model was applied. When *P* was ≥0.10 or *I*^2^ was ≤ 50%, a fixed-effects model was used. No subgroup analyses were planned or performed.

MDs were estimated from medians, ranges, or interquartile ranges using methods described by Shi et al. (22, 23). The study characteristics are summarized in Table 2. The forest plots (Figures 2–5) display individual and pooled effect sizes.

**Table 2:** Associations between inflammatory indicators and PSD.

References	Diseases	Sources of cases	Number of cases	Genders		Age	Depression evaluation Scale	Clinical associations
				Male	Female			
Su et al. (24)	AIS	China	318	217 (68.24%)	101 (31.76%)	63 (57–71)	HAMD-17>7	SII levels correlate with PSD risk, and the highest SII tertile is significantly and independently associated with increased PSD risk
Chen et al. (25)	IS	China	299	195 (65.2%)	104 (34.8%)	62.77 ± 10.15	HAMD-17 > 7	Patients with PSD exhibit significantly higher NLR levels than those without PSD; furthermore, gender is associated with PSD, with women being more susceptible to the condition
Huang et al. (26)	AIS	China	363	233 (64.2%)	130 (35.8%)	62.6 ± 10.1	HAMD-17 > 7	Compared with NPSD patients, PSD patients were predominantly female; moreover, PLR was significantly and independently associated with PSD risk
Hu et al. (27)	AIS	China	376	224 (59.57%)	152 (40.43%)	61.37 ± 10.34	HAMD-17 > 7	Patients with PSD exhibited significantly higher NLR and PLR levels compared to those without PSD; compared to the non-PSD group, PSD patients were predominantly female. NLR and PLR levels in PSD patients were significantly higher than those in the NPSD group, while LMR levels were lower but not significantly different
Han et al. (28)	Chronic stroke	China	200	142 (71%)	58 (29%)	< 60 54.50% ≥ 60 45.50%	HAMD-24 ≥ 8	Furthermore, both NLR and PLR were independently associated with PSD: each unit increase in NLR was associated with a 2.18-fold increase in PSD risk, and each 10-unit increase in PLR was associated with a 31.7% increase in PSD risk
Hu et al. (29)	IS	China	432	272 (64.3%)	160 (35.7%)	62.58 ± 10.27	HAMD-17 > 7	SII, PLR, and NLR levels are elevated in patients with PSD, and an SII value >547.30 is independently associated with the incidence of PSD
Chen et al. (30)	Stroke	China	103	72 (69.9%)	31 (30.1%)	57 (53–67)	DSM-V	Patients with PSD exhibited higher NLR and PLR levels compared to those with NPSD, while LMR levels showed no significant difference
Xiao et al. (31)	AIS	China	332	155 (46.70%)	177 (53.30%)	63.12 ± 7.25	HAMD-17 > 7	SII levels are an independent risk factor for PSD in AIS patients
Ding et al. (32)	AIS	China	203	138 (67.98%)	65 (32.02%)	66.58 ± 10.68	DSM-IV	LMR is an independent risk factor for PSD and is significantly associated with PSD at 3 months

**Figure 2:**
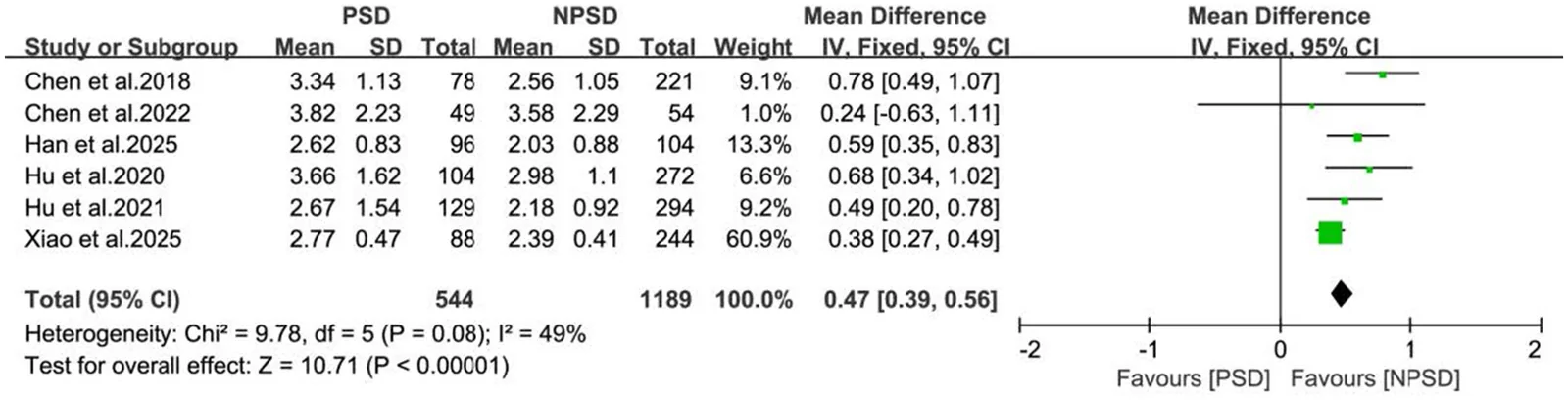
Forest plot for the differences in serum NLR between the PSD group and NPSD group.

## 3 Effects of combined inflammatory markers on the alteration and diagnostic value of PSD

The initial search yielded 426 records (PubMed: 39; Google Scholar: 200; Web of Science: 135; CNKI: 52). After removing duplicates, 174 records were screened by title and abstract. Of these, 159 were excluded because they were not cohort studies, did not report relevant inflammatory markers, or were reviews, case reports, or conference abstracts. Fifteen full-text articles were assessed for eligibility, of which 9 were ultimately included in the systematic review (24–32). The screening process is detailed in the PRISMA flow diagram (Figure 1). This analysis included a total of 2,626 patients, among whom 763 developed PSD, while 1,863 did not. The participants included 1,648 males and 978 females, with a female proportion of 37.24%. Associations between the NLR, PLR, LMR, and SII and PSD were evaluated in 6, 6, 4, and 3 studies, respectively. These studies diagnosed various types of PSD, including 5 studies on AIS patients with depression, 2 on IS patients with depression, 1 on chronic stroke patients with depression, and 1 on stroke patients with depression. Diagnostic tools for PSD included the 17-item Hamilton Depression Scale (HAMD), 18-HAMD, Diagnostic and Statistical Manual of Mental Disorders-IV criteria (DSM-IV), and DSM-V (Table 2). Associations between the NLR, PLR, LMR, and SII and PSD were assessed in 6, 6, 4, and 3 studies, respectively. The NLR, PLR, and SII were higher in PSD patients than in non-PSD patients, while the LMR was lower.

### 3.1 Risk of bias assessment

The NOS scores for the nine included studies ranged from 8 to 9, with all the studies rated as high quality (scores ≥7). Detailed scores for each study across the three domains (selection, comparability, and outcome) are presented in Table 1.

### 3.2 Value of the NLR in predicting PSD

A total of 6 studies involving 544 PSD patients and 1,189 NPSD patients were included. There was mild heterogeneity among the related studies (*P* = 0.08, *I*^2^ = 49%), and a fixed-effects model was used. The results revealed that the serum NLR in PSD patients was significantly greater than that in NPSD patients (*MD* = 0.47, 95% *CI* = [0.39, 0.69], *P* < 0.00001; Figure 2). An analysis of the 6 included studies revealed that Han et al. (28) enrolled chronic stroke patients, whereas the other 5 studies included AIS patients. The inclusion of different types of stroke patients with concurrent depression further validated that an elevated NLR can be used to independently predict PSD. Hu et al. (27) suggested that a higher NLR is associated with depression at 6 months post-stroke. Among these studies, Chen et al. (25) and Hu et al. (27) reported that sex is associated with PSD, primarily in females. On the basis of these findings, in this study, we speculated that the NLR could serve as an independent inflammatory marker for predicting PSD, and there may be sex differences.

### 3.3 Value of the PLR in predicting PSD

A total of 6 studies involving 543 PSD patients and 1,254 NPSD patients were included in the analysis. The PLR values for each study are shown in Table 2. Significant heterogeneity was observed among the related studies (*P* < 0.00001, *I*^2^ = 90%), and a random-effects model was applied. The results indicated that the PLR in the serum of PSD patients was significantly greater than that in the serum of NPSD patients (*MD* = 20.79, 95% *CI* = [12.25, 29.33], *P* < 0.00001; Figure 3). An analysis of these 6 studies revealed that unlike the other 5 studies, which included AIS patients, Han et al. (28) reported that the PLR was independently associated with PSD in chronic stroke patients. The inclusion of different types of stroke patients with concurrent depression further validated that an elevated PLR can be used to independently predict PSD. Among these, the findings of Hu et al. (27) suggested that sex is associated with PSD, primarily in females. On this basis, the present study hypothesizes that the PLR may serve as an independent inflammatory marker for predicting PSD and that there may be sex differences.

**Figure 3:**
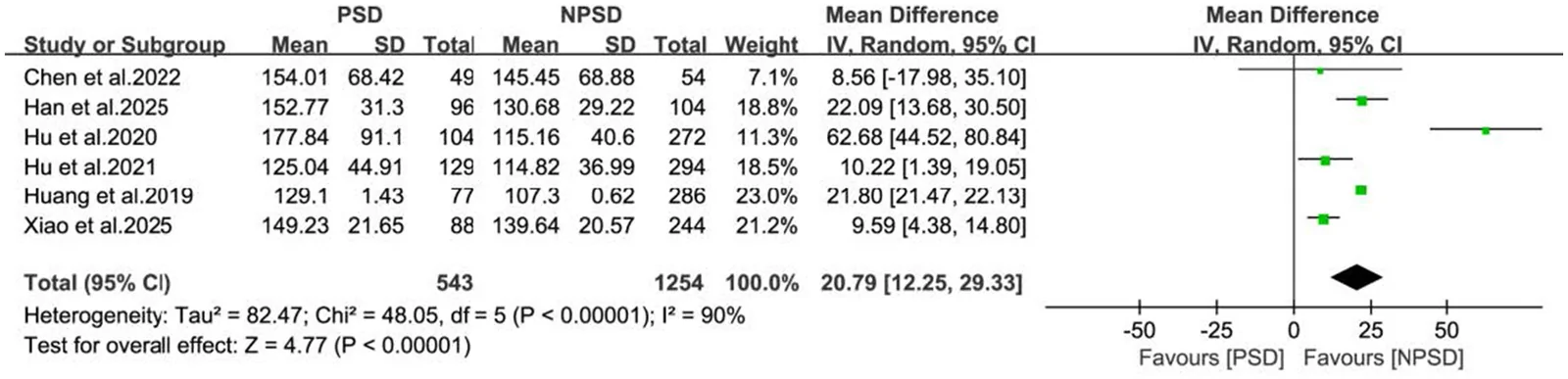
Forest plot for the differences in serum PLR levels between the PSD group and NPSD group.

### 3.4 Value of LMR in predicting PSD

The analysis included a total of 4 studies involving 277 PSD patients and 561 NPSD patients. The LMR values for each study are presented in Table 2. There was moderate heterogeneity among the related studies (*P* = 0.04, *I*^2^ = 63%), and a random-effects model was employed. The results indicated that the serum LMR was significantly lower in PSD patients than in NPSD patients (*MD* = −0.21, 95% *CI* = [−0.34, −0.08], *P* = 0.001; Figure 4). However, on the basis of the results of these 4 studies, with the exceptions of Ding et al. (32), who reported that the LMR was significantly different between PSD and NPSD patients and that the LMR was independently associated with PSD at 3 months, the other three studies reported that the LMR differed between PSD and NPSD patients but lacked statistical significance and thus could not be considered associated with the risk of PSD occurrence. This discrepancy may be related to the time elapsed after disease onset.

**Figure 4:**
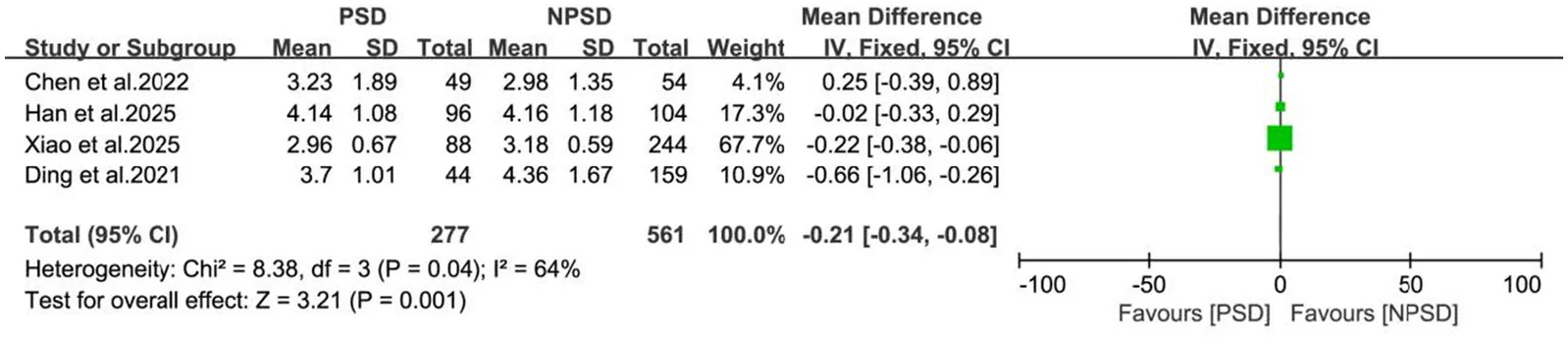
Forest plot for the differences in serum LMR between the PSD group and the NPSD group.

### 3.5 Value of the SII in predicting PSD

The analysis included a total of 3 studies, comprising 315 PSD patients and 758 NPSD patients. The SII values for each study are presented in Table 2. There was moderate heterogeneity among the related studies (*P* = 0.04, *I*^2^ = 70%), and a random-effects model was employed. The results indicated that the serum SII was significantly greater in PSD patients than in NPSD patients (*MD* = 123.92, 95% *CI* = [68.85, 178.98], *P* < 0.0001; Figure 5). All three studies concluded that the SII is independently associated with the risk of PSD, and Hu et al. (29) suggested that an SII >547.30 is independently associated with the incidence of PSD. However, all three studies included only IS patients, with the majority being AIS patients; thus, only the association between the SII and depression in IS patients was represented. It remains unclear whether this association applies to other types of stroke patients with comorbid depression.

**Figure 5:**
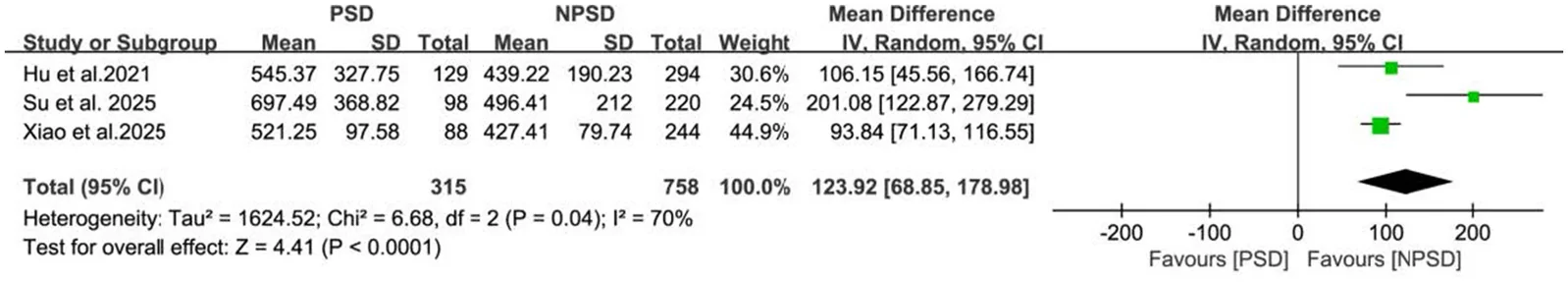
Forest plot for the differences in serum SII values between the PSD group and the NPSD group.

## 4 Mechanisms by which peripheral immune cells influence PSD

The results of the analysis of the relevant literature included in this study revealed that the abnormal expression of immune cell subsets in peripheral blood is closely related to the risk of PSD occurrence. High levels of neutrophils, platelets, and monocytes and low levels of lymphocytes are associated with an increased risk of PSD. Furthermore, multiple studies have confirmed that peripheral immune cells can play a regulatory role in the occurrence of PSD through systemic inflammatory responses (Figure 6).

**Figure 6:**
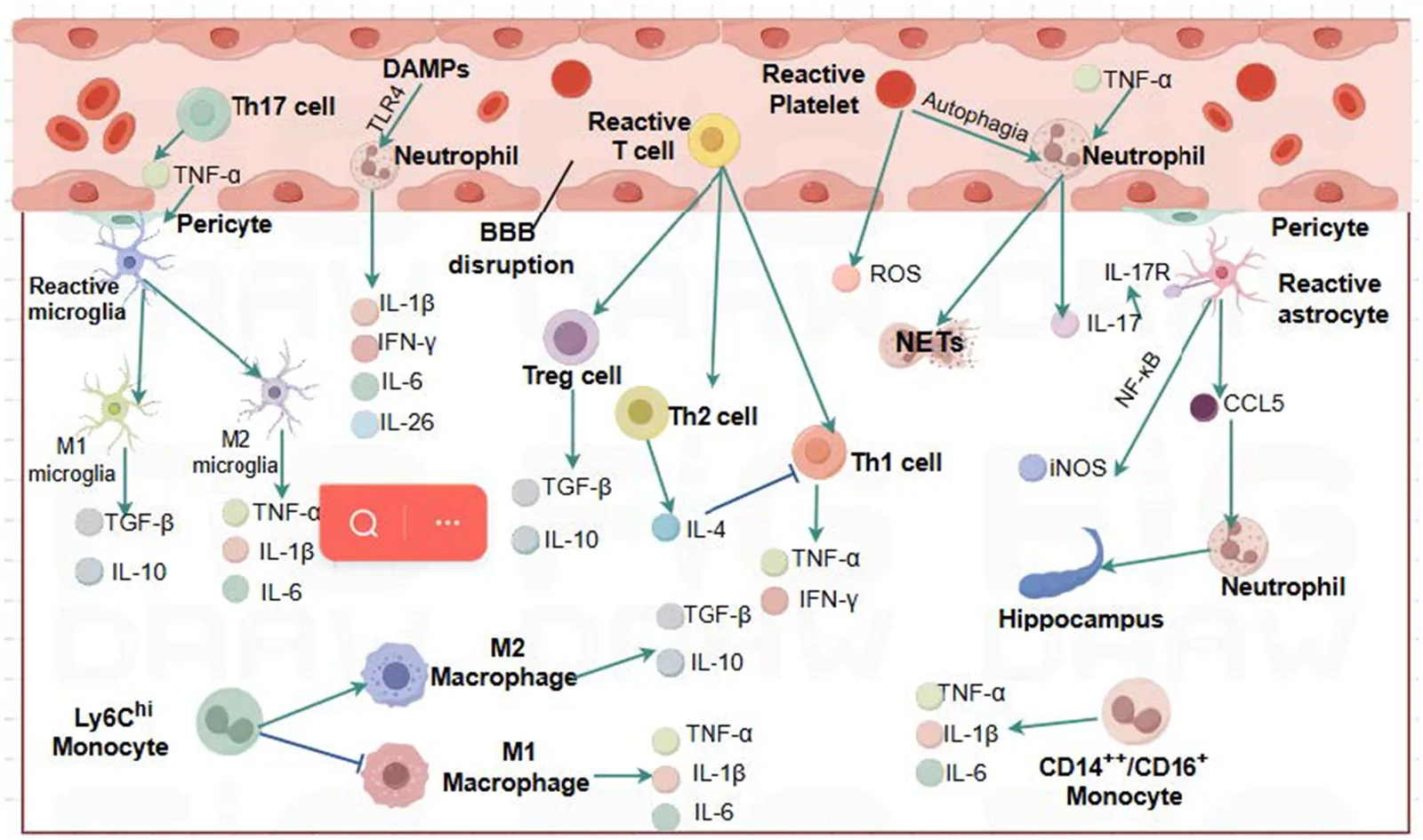
Mechanistic diagram of peripheral immune cells in PSD. This image illustrates the alterations and potential mechanisms of peripheral immune cells in PSD. Ischemia and hypoxia can lead to cellular damage; the release of injury-associated signaling patterns; and the activation of neutrophils, T cells, microglia, astrocytes, platelets, and monocytes/macrophages. These immune cells collectively construct a PSD inflammatory microenvironment, exacerbating brain damage and depressive states in PSD patients (created by figdraw).

### 4.1 Neutrophil

Neutrophils are peripheral immune cells that infiltrate brain tissue early after stroke. In various central nervous system injury diseases, such as AIS, cerebral hemorrhage, traumatic brain injury, and spinal cord injury, early infiltration of neutrophils into damaged brain tissue can occur. These neutrophils produce cytotoxic molecules such as proinflammatory factors, matrix metalloproteinases, nitric oxide (NO), and ROS, thereby accelerating brain injury (33). With respect to the activation mechanism, poststroke ischemia leads to cellular damage, releasing damage-associated molecular patterns that activate pattern recognition receptors such as TLR4, triggering neutrophil activation. Once activated, neutrophils release large amounts of IL-1β, IL-6, IL-17, IFN-γ, and IL-26 (34). Studies have shown that TLR4 activates the downstream NF-κB signaling pathway, initiating the transcription of proinflammatory cytokines such as TNF-α, IL-1β, and IL-6. After binding to specific receptors, these proinflammatory factors activate downstream signal transduction, which not only induces the production of inflammatory mediators but also transmits death signals, promoting cell death and exacerbating PSD (35). Research has shown that IL-17 can bind to upregulated IL-17 receptors on the surfaces of neurons, astrocytes, and microglia, recruiting the Act1 protein and activating signaling pathways such as the NF-κB and GSK3β pathways to induce the expression of proinflammatory cytokines, chemokines, and iNOS, thereby exacerbating neuroinflammation. IL-17 acts on cerebrovascular endothelial cells, disrupting BBB integrity through NADPH oxidase-dependent endothelial contraction and the downregulation of tight junction proteins. Additionally, it increases ICAM-1 expression in endothelial cells, promoting the transendothelial migration of neutrophils and monocytes, which worsens parenchymal brain injury. IL-17 synergizes with TNF-α to stimulate astrocytes to release CXCL1, recruiting neutrophils and prolonging the inflammatory response (36).

Stroke activates neutrophils, causing them to aggregate in the brain and leading to the release of nuclear and granular contents, forming a reticular structure with deoxyribonucleic acid as the skeleton and adhering to histones and granular proteins, known as neutrophil extracellular traps (NETs) (37). NETs release various toxic proteases, inducing endothelial cell damage while increasing vascular permeability. Like AIS, NETs play a “double-edged sword” role. On the one hand, NETs can act as a barrier to limit the spread of pathogens (bacteria, fungi, viruses, parasites, etc.) and the diffusion of inflammatory factors, preventing damage to normal tissues; on the other hand, they play a significant role in the thrombus formation process, participating in the scaffold of thrombus formation and maintaining its stability. Excessive formation of intravascular reticular structures is also an important cause of chronic inflammatory responses (38). Studies have confirmed that NETs can regulate poststroke brain injury. For example, Liu et al. (39) reported that neutrophils release NETs after activation. Stroke increases BBB permeability, providing a pathway for peripheral NETs to infiltrate the brain parenchyma, while NETs further exacerbate BBB damage. Lcn2 released by NETs triggers abnormal Ca^2+^ signaling in astrocytes, leading to morphological remodeling and functional activation. Activated astrocytes mediate mood disorders such as depression by altering the neuroimmune microenvironment.

Additionally, Yao et al. (40) reported that under depressive conditions, hippocampal astrocytes highly express CCL5; the expression of CCR5 receptors on neutrophil surfaces is upregulated, and these cells bind to CCL5 secreted by astrocytes and are subsequently chemotactically recruited to the hippocampus. Moreover, chronic stress increases BBB permeability, providing conditions for neutrophil infiltration. Neutrophils infiltrating the hippocampus are in an activated state, with enhanced phagocytic capacity, directly engulfing neuronal synapses, leading to reduced synaptic density in the hippocampus, impaired synaptic transmission, and decreased neuronal excitability, resulting in a depressive state.

These studies indicate that neutrophils play a significant role in stroke-related complications: by releasing inflammatory factors and interacting with glial cells, they exacerbate neuroinflammation and disrupt the BBB, thereby worsening parenchymal damage in PSD patients. Simultaneously, through oxidative stress and NET binding to astrocytes, they impair hippocampal function in stroke patients, causing a depressive state and thus aggravating PSD.

### 4.2 Lymphocytes

Lymphocytes play a dual regulatory role in the pathological process of stroke, with significant functional heterogeneity among different lymphocyte subsets. T cells can initiate immune responses such as Th1 and Th17 responses, release large amounts of proinflammatory cytokines and mediate systemic inflammatory reactions in the early stages of stroke, whereas regulatory T cells (Tregs) secrete anti-inflammatory cytokines to exert anti-inflammatory effects (41). Naive T cells typically do not cross the BBB. After stroke occurs, immune cells with antigen-presenting functions in the central nervous system (such as microglia) are activated, releasing proinflammatory cytokines such as TNF-α, IL-6, and IL-1β. By increasing the expression levels of adhesion molecules, they promote the recruitment of peripheral T cells into the brain (42). Research has shown that CD4^+^ and CD8^+^ T lymphocytes are key participants in the pathological process of IS. By releasing proinflammatory cytokines, they induce the expression of adhesion molecules in cerebral microvascular endothelial cells and activate brain-resident macrophages and other leukocyte subsets, collectively constructing a proinflammatory microenvironment. Additionally, T cells promote a pro-thrombotic phenotype in postischemic cerebral venules, increase the adhesion and aggregation of leukocytes and platelets on vascular walls, expand infarct volume, and exacerbate neurological deficits (43).

Furthermore, the results of this study revealed that patients with varying severity of depression exhibit abnormalities in CD4+ T lymphocyte subsets and related cytokines. Specifically, Th1 cells influence brain neuronal function and neurotransmitter balance through the secretion of proinflammatory factors such as IL-2, IFN-γ, and TNF-α, thereby inducing depression. Th17 cells increase neuroinflammation or neuronal death by secreting proinflammatory factors and activating microglia and astrocytes, thereby increasing susceptibility to depression. They also directly or indirectly affect the immune environment, exacerbating oxidative stress and the excitotoxicity of microglia and disrupting the BBB, and their overactivation is associated with depressive symptoms. Treg cells express Foxp3, CD4, and CD25 and secrete anti-inflammatory factors such as IL-10 and TGF-β, maintaining immune homeostasis and self-tolerance. Their reduced numbers lead to the formation of an inflammatory environment in the brain, triggering or exacerbating chronic depressive behaviors and contributing to immune dysregulation. Moreover, the results of this study revealed that IL-4 derived from Th2 cells can inhibit the production of IFN-γ and IL-2 by Th1 cells. In depressive states, overactivation of the hypothalamic–pituitary–adrenal (HPA) axis suppresses IL-4 secretion. IL-6 reduces neurotransmitter levels, activates systemic immune-inflammatory responses, modulates HPA axis activity, and exacerbates depression. Additionally, IL-6 and TGF-β jointly regulate Th17 and Treg cell differentiation (high levels of IL-6 promote Th17 differentiation and inhibit Treg differentiation) and participate in immune imbalance (44).

In summary, lymphocytes play a dual role in the pathophysiological process of PSD. On the one hand, proinflammatory subsets (CD4+ T cells, CD8+ T cells, Th1 cells, and Th17 cells) release proinflammatory factors, resulting in the cerebral inflammatory microenvironment in PSD patients and exacerbating damage and depressive-like behaviors. On the other hand, Treg cells secrete anti-inflammatory factors, maintaining immune homeostasis and exerting protective effects.

### 4.3 Platelet

Platelets are key in the pathophysiology of stroke; they interact with immune cells to release inflammatory factors, disrupt the BBB, and exacerbate ischemia–reperfusion injury (45). Gao et al. (46) reported that activated platelets release HMGB1-containing platelet microvesicles, inducing neutrophil autophagy, promoting NET formation, and aggravating stroke injury. Simultaneously, NET surfaces highly express tissue factors, binding coagulation factors and activating platelets, increasing thrombin–antithrombin complex levels, promoting fibrin formation, enhancing cerebral thrombosis in AIS patients, and worsening brain damage. Kleinschnitz et al. (47) reported that platelets bind to the endothelial cell surface von Willebrand factor via the GPIb receptor, achieving initial adhesion to hypoxic cerebral endothelial cells. During ischemia, the subendothelial matrix is exposed, and collagen binds to the platelet surface receptor GPVI, triggering tyrosine phosphorylation and Ca^2+^ mobilization, inducing platelet morphological changes, and promoting firm platelet attachment to vessel walls, subsequently activating platelets, ultimately promoting cerebral thrombosis and aggravating ischemic brain injury. Wu et al. (48) reported that poststroke platelets interact with immune cells such as neutrophils, inducing proinflammatory factor release and exacerbating cerebral inflammation. After platelet activation, the mitochondrial membrane potential decreases, and ROS production increases, further amplifying activation signals. Through the induction of BV2 microglial polarization toward a proinflammatory phenotype and the upregulation of iNOS and COX-2 expression via the p-JNK/p38 pathway, cerebral inflammation intensifies. By participating in thrombo-inflammatory responses, they reduce tight junction protein expression, increase BBB permeability, and aggravate brain damage.

Williams et al. (49) reported that platelets are the primary transporters of serotonin in the body, taking up serotonin through serotonin transporters and storing it in dense granules containing calcium and adenosine triphosphate; after serotonin is released, it binds to 5-HT2A receptors on the platelet membrane, which can induce further platelet activation and vasoconstriction. Depression is associated with weakened serotonin signaling in the central nervous system, potentially leading to the upregulation of platelet 5-HT2A receptor density through a negative feedback loop. Recent studies have shown that the activity of arginase II in the platelets of patients with major depressive disorder (MDD) is significantly increased, which can inhibit nitric oxide (NO) synthesis and disrupt NO-mediated platelet inhibition. The expression of p47phox, a regulatory subunit of NADPH oxidase 2 in platelets, is upregulated, promoting the generation of reactive oxygen species (ROS), leading to increased oxidative damage and exacerbating platelet aggregation, adhesion, and recruitment (50). García-Fernández et al. (51) reported that thrombospondin-2 is involved in platelet activity regulation, vascular function maintenance, and neuroinflammatory processes. Its elevation increases the risk of depression-related suicide. Platelet-derived growth factor BB (PDGF-BB) promotes pericyte migration and stabilization in the brain, regulates neurogenesis, and reverses depressive-like behaviors in hippocampal circuits. Reduced PDGF-BB levels increase the risk of suicide attempts among depressed patients. As a platelet-associated immune marker, decreased serotonin levels contribute to the pathological processes underlying depression severity by affecting neurotransmitter balance.

In summary, platelets are key mediators of poststroke thrombosis and neuroinflammation, exacerbating poststroke brain injury through mechanisms such as activation and aggregation, regulation of signaling pathways, and interaction with immune cells. Studies have confirmed that platelets aggravate the severity of depression in patients through remodeling of the 5-HT system, excessive activation with proinflammatory/coagulant phenotypes, disruption of signaling pathways, and release of bioactive substances, all of which play crucial roles in the development and progression of PSD.

### 4.4 Monocyte

Monocytes, as crucial peripheral immune cells, play a significant role in the pathological progression of stroke. Monocytes can differentiate into cells with potential phagocytic functions in ischemic brain tissue, namely, monocyte-derived macrophages (MDMs) (52). Initially, the presence of these cells was considered a marker of exacerbated inflammatory responses; however, recent studies suggest that these cells play a more complex role in the pathological process of AIS. Ly6C^hi^ monocytes exhibit protective effects on AIS by promoting macrophage polarization toward the M2 phenotype; increasing the expression of M2 markers such as Ym1, Arg1, and IL-10; and inhibiting M1 macrophage polarization and reducing peroxynitrite production (53). CCR2 deficiency prevents monocytes from migrating from the spleen to the periphery, thereby reducing brain infiltration, lowering the expression of angiogenic factors (VEGF and Ang-1), and delaying repair (54). After stroke, CCR2+ inflammatory monocytes are key regulators of cerebrovascular repair and functional recovery. Choi et al. (55) reported that after stroke, monocytes infiltrate the brain injury area and release IL-6. IL-6 binds to IL-6Ra on the surface of microglia, promoting microglial differentiation into the RAMs subset via the IL-6/JAK/STAT3 signaling pathway. RAMs secrete factors such as VEGA, CD24, and CD44, directly driving vascular repair and improving poststroke cognitive and motor functions. Wattanit et al. (56) reported that poststroke monocytes are recruited to the injured cerebral hemisphere through the binding of the CCR2 receptor to its ligand CCL2 and that CCR^2+^ monocytes differentiate into MDMs in the circulation. Poststroke MDMs exist in two subsets: The anti-inflammatory phenotype highly expresses anti-inflammatory genes such as TGF, CD163, Ym1, CXCL13, and CCL22, which participate in inflammation resolution. Blocking monocyte recruitment significantly reduces anti-inflammatory gene expression, disrupts inflammation resolution, and leads to long-term functional impairment (56).

Additionally, Hasselmann et al. (57) reported that specific phenotypic shifts occur in monocyte subsets of MDD patients. The relative frequency of classical monocytes (CD14^++^/CD16^−^) decreases, whereas the absolute count of intermediate monocytes (CD14^++^/CD16^+^) increases. The frequency and absolute count of non-classical monocytes (CD14^+^/CD16^++^) are significantly elevated and associated with abnormalities in steroid signaling pathways. The proportion of classical monocytes expressing IL-1β increases, intermediate monocytes show elevated proportions of IL-1β and IL-6 expression but reduced IL-10 expression, and the number of cells producing TNF-α, IL-1β, IL-6, and IL-10 significantly increases. Non-classical monocytes exhibit increased IL-1β expression and reduced IL-6 expression, collectively driving a systemic proinflammatory state in MDD patients, with significantly elevated serum TNF-α and IL-1β levels. Concurrently, the expansion of intermediate monocytes and increased production of proinflammatory cytokines (IL-1β and IL-6), along with abnormal IL-1β expression in classical and non-classical monocytes, exacerbate systemic inflammation, thereby contributing to the pathogenesis of MDD (58). Furthermore, Oh et al. (59) reported that activated microglia in depressed patients release MCP-1, which binds to CCR2 on peripheral monocytes, guiding their migration along chemokine gradients toward the BBB. BBB endothelial cells express ICAM-1 and VCAM-1, facilitating monocyte adhesion, rolling, and extravasation across the BBB. Monocytes entering the brain secrete proinflammatory cytokines such as IL-1β and TNF-α, inducing neuronal excitotoxicity, reducing serotonin tone, and ultimately triggering depression. Additionally, the binding of MCP-1 to CCR2 on brain microvascular endothelial cells promotes the phosphorylation of myosin light chains and ezrin/radixin/moesin proteins, disrupting BBB tight junction integrity and further enhancing monocyte recruitment into the brain.

In summary, monocytes in PSD can promote brain injury repair by releasing anti-inflammatory factors; on the other hand, their subgroup imbalance in PSD, combined with activated microglia, allows them to cross the BBB into the brain, disrupting the integrity of the BBB and ultimately inducing depression.

## 5 Challenges and prospects

Although all included studies were rated as high quality (NOS scores 8–9), the observational nature of cohort designs and lack of multicenter data limit the overall evidence. Residual confounding cannot be completely excluded, as some studies did not adjust for sex or age in their multivariate models.

An analysis of 9 studies revealed that elevated ratios of NLR, PLR, and SII, along with a reduced LMR for peripheral blood composite inflammatory markers, are significantly associated with an increased risk of PSD. Among these factors, the NLR and PLR can independently predict depression following different types of stroke, whereas the SII has independent predictive value for PSD after IS. Additionally, the predictive effects of the NLR and PLR may differ across the sexes. These findings are highly consistent with those of previous research: Wang et al. (60) conducted a cross-sectional study using a database and concluded that the SII is significantly positively correlated with PSD; Chong et al. (61) confirmed that a reduced LMR is independently associated with a higher risk of PSD.

Further investigation into the pathological mechanisms through which peripheral immune cells mediate PSD revealed that neutrophils can exacerbate neuroinflammation and depressive states by releasing inflammatory factors, forming NETs, and impairing hippocampal function. Lymphocyte subsets have dual effects on PSD: proinflammatory subsets create a proinflammatory microenvironment in the brain, worsening depression, whereas anti-inflammatory subsets secrete anti-inflammatory factors to maintain immune homeostasis. Platelets play a key role in poststroke thrombosis and neuroinflammation and exacerbate depressive severity through 5-HT. Monocytes have both damaging and reparative effects on PSD, and an imbalance in their subset numbers can intensify inflammatory infiltration and induce depression. Various immune cells collectively mediate the occurrence and progression of PSD through interactions, BBB infiltration, and the regulation of neuroinflammation.

This study confirms the central role of inflammatory responses in the pathophysiological process of PSD; clarifies the potential value of the NLR, PLR, LMR, and SII as inflammatory markers for PSD; and elucidates the key mechanisms through which peripheral immune cells regulate PSD, providing a basis for early risk assessment of PSD. However, current related research still has limitations. First, the diagnostic or predictive performance of these inflammatory markers could not be formally evaluated in this review, as most included studies reported only adjusted odds ratios without providing sensitivity, specificity, or AUC values; the limited available data are summarized in Tables 3, 4. Second, existing studies focus primarily on IS (especially AIS) as the research subject, with fewer studies on hemorrhagic stroke and mixed stroke and limited research on the acute and recovery phases of stroke, making determining the predictive value of these four indicators for PSD in different types and courses difficult. Third, only one study provides a reference cutoff value for the SII, while other indicators lack unified diagnostic thresholds; thus, we rely solely on comparisons of the levels between PSD patients and NPSD patients. Fourth, most of the included studies were retrospective in design, and all were conducted in China, which may limit the generalizability of our findings to other populations and healthcare settings.

**Table 3:** Diagnostic and predictive performance metrics reported in the included studies.

References	Inflammatory marker	Analysis type	Cutoff value	AUC (95% CI)	Sensitivity	Specificity	Adjusted OR (95% CI)	*P*-value
Chen et al. (25)	NLR	Logistic regression	≥3.701	NR	NR	NR	4.038 (2.174–7.500)	< 0.001
Huang et al. (26)	PLR	Logistic regression (tertile 3 vs. tertile 1)	>127.92	NR	NR	NR	5.154 (1.933–13.739)	0.001
Hu et al. (27)	NLR	Logistic regression (quartile 4 vs. quartile 1–3)	≥4.02	0.726 (0.643–0.809)	NR	NR	3.926 (2.365–7.947)	< 0.001
Hu et al. (27)	PLR	Logistic regression (quartile 4 vs. quartile 1–3)	≥203.74	0.701 (0.622–0.780)	NR	NR	3.853 (2.214–6.632)	0.002
Ding et al. (32)	MLR	Logistic regression	0.2298	0.615 (0.524–0.705)	0.773	0.541	18.020 (1.127–288.195)	0.041
Hu et al. (29)	SII	Logistic regression (tertile 3 vs. tertile 1)	>547.30	NR	NR	NR	2.181 (1.274–3.732)	0.004
Hu et al. (29)	NLR	Logistic regression	NR	NR	NR	NR	1.728 (1.009–2.958)	0.046
Hu et al. (29)	PLR	Logistic regression	NR	NR	NR	NR	1.822 (1.063–3.122)	0.029
Chen et al. (30)	Monocyte count	Logistic regression	NR	NR	NR	NR	0.057 (0.005–0.686)	0.024
Chen et al. (30)	CRP rs2794520 C/T	Logistic regression	NR	NR	NR	NR	3.364 (1.039–10.898)	0.043
Han et al. (28)	NLR	Logistic regression	NR	NR	NR	NR	2.181 (1.453–3.274)	< 0.001
Han et al. (28)	PLR	Logistic regression	NR	NR	NR	NR	1.028 (1.015–1.040)	< 0.001
Su et al. (24)	SII	Logistic regression (tertile 3 vs. tertile 1)	602.5	0.659 (0.592–0.726)	0.582	0.7	3.502 (1.582–7.752)	0.002
Xiao et al. (31)	SII	Logistic regression	NR	0.881 (0.843–0.918)	0.852	0.783	1.007 (1.003–1.010)	< 0.001

**Table 4:** Summary of diagnostic performance.

Marker	Number of studies	AUC range	Cutoff range	Sensitivity range	Specificity range
NLR	3	0.726 (1 study)	3.70–4.02	NR	NR
PLR	3	0.701 (1 study)	127.93–203.74	NR	NR
MLR/LMR	2	0.615 (1 study)	0.2298	0.773	0.541
SII	4	0.659–0.881	547.30–602.50	58.2%−85.2%	70.0%−78.3%

NR, not reported.

The substantial heterogeneity for the PLR (*I*^2^ = 90%) and SII (*I*^2^ = 70%) warrants consideration. For the PLR, possible sources include variations in absolute PLR values across studies, with Huang et al. (26) reporting a median PLR of 107.3 in non-PSD patients, while Hu et al. (27) reported a mean of 177.8 in PSD patients; differences in PSD assessment timing, ranging from admission (26) to 1 month (25, 29) and 6 months post-stroke (27); and stroke subtypes, with most studies focusing on AIS, whereas Han et al. included chronic stroke patients. For the SII, heterogeneity may arise from different cutoff values (>547.30 vs. >602.50) (24, 29) and follow-up durations (1 month vs. 3 months) (24, 29). These factors should be considered when pooled estimates are interpreted, and standardized study designs are needed in future research.

With respect to clinical translation, several barriers remain before the NLR, PLR, LMR, and SII can be incorporated into routine practice. First, validated cutoff values are lacking for most markers, with only the SII having been examined in a limited number of studies (cutoffs ranging from 547.30 to 602.50) (24, 29). Second, prospective validation in independent cohorts is needed to confirm their predictive ability. Third, the optimal timing of biomarker measurement and the optimal combination with other clinical predictors remain to be determined. These gaps must be addressed before these biomarkers can be integrated into routine clinical practice for PSD risk stratification.

On this basis, future research could focus on hemorrhagic stroke, mixed stroke, and stroke patients at different disease stages to clarify the predictive specificity and optimal cutoff values of the NLR, PLR, LMR, and SII for different types of stroke, further improving the early prediction system for PSD and enhancing the accuracy of early clinical identification and intervention.
