# Supplementary material for: Predictive roles of inflammatory indicators (NLR, PLR, LMR and SII) in poststroke depression rehabilitation: a systematic review
Source: Front Neurol. 2026 Oct 5;17:1890435. doi: 10.3389/fneur.2026.1890435 (PMC13677359; doi:10.3389/fneur.2026.1890435)
Supplement: Supplementary file 1 [file Supplementary_File_1.docx]

Supplementary Tables

Supplementary Table S1. Detailed search strategies for each database

| **Database** | **Search Query** | **Notes** |
| --- | --- | --- |
| PubMed | ((("neutrophil-to-lymphocyte ratio"[Title/Abstract] OR "NLR"[Title/Abstract]) OR ("platelet-to-lymphocyte ratio"[Title/Abstract] OR "PLR"[Title/Abstract]) OR ("lymphocyte-to-monocyte ratio"[Title/Abstract] OR "LMR"[Title/Abstract]) OR ("systemic immune-inflammation index"[Title/Abstract] OR "SII"[Title/Abstract])) AND ("poststroke depression"[Title/Abstract] OR "PSD"[Title/Abstract] OR "depression after stroke"[Title/Abstract] OR "poststroke depression"[Title/Abstract])) | Search fields: Title/Abstract; Date: January 31, 2026 |
| Web of Science | ("neutrophil-to-lymphocyte ratio" OR NLR OR "platelet-to-lymphocyte ratio" OR PLR OR "lymphocyte-to-monocyte ratio" OR LMR OR "systemic immune-inflammation index" OR SII) AND ("poststroke depression" OR PSD OR "depression after stroke" OR poststroke depression) | Search field: Topic (TS); Date of search: January 31, 2026; No language or publication type restrictions applied |
| Google Scholar | ("neutrophil-to-lymphocyte ratio" OR NLR OR "platelet-to-lymphocyte ratio" OR PLR OR "lymphocyte-to-monocyte ratio" OR LMR OR "systemic immune-inflammation index" OR SII) AND ("poststroke depression" OR PSD) | Limited to first 200 records sorted by relevance due to search engine constraints; Date: January 31, 2026 |
| CNKI (China National Knowledge Infrastructure) | SU=('中性粒细胞与淋巴细胞比值' + 'NLR' + '血小板与淋巴细胞比值' + 'PLR' + '淋巴细胞与单核细胞比值' + 'LMR' + '全身免疫炎症指数' + 'SII') AND SU=('卒中后抑郁' + 'PSD' + '脑卒中后抑郁' + '中风后抑郁') | Search field: Topic includes title, abstract, and keywords; Language: Chinese; Date: January 31, 2026 |

Supplementary Table S2. PRISMA 2020 main checklist (27 items)

| **Section and Topic** | **Item#** | **Checklist item** | **Location where item is reported** |
| --- | --- | --- | --- |
| **TITLE** |  |  |  |
| Title | 1 | Identify the report as a systematic review | Page 1, 3 |
| **ABSTRACT** |  |  |  |
| Abstract | 2 | See the PRISMA 2020 for Abstracts checklist. | Table S2 |
| **INTRODUCTION** |  |  |  |
| Rationale | 3 | Describe the rationale for the review in the context of existing knowledge. | Page 2,3 |
| OBjectives | 4 | Provide an explicit statement of the objective(s) or question(s) the review addresses. | Page 3 |
| **METHODS** |  |  |  |
| Eligibility criteria | 5 | Specify the inclusion and exclusion criteria for the review. | Page 3, 4 |
| Information sources | 6 | Specify all databases, registers, websites, organisations, reference lists and other sources searched or consulted to identify studies. Specify the date when each source was last searched or consulted. | Page 3 |
| Search strategy | 7 | Present the full search strategies for all databases, registers and websites, including any filters and limits used. | Page 3, Supplementary Table S1 |
| Selection process | 8 | Specify the methods used to decide whether a study met the inclusion criteria of the review, including how many reviewers screened each  record and each report retrieved, whether they worked independently, and if applicable, details of automation tools used in the process. | Page 4 |
| Data collection process | 9 | Specify the methods used to collect data from reports, including how many reviewers collected data from each report, whether they worked  independently, any processes for obtaining or confirming data from study investigators, and if applicable, details of automation tools used in the process. | Page 4 |
| Data items | 10a | List and define all outcomes for which data were sought. Specify whether all results that were compatible with each outcome domain in each study were sought (e.g. for all measures, time points, analyses), and if not, the methods used to decide which results to collect. | Page 4 |
|  | 10b | List and define all other variables for which data were sought (e.g. participant and intervention characteristics, funding sources). Describe any assumptions made about any missing or unclear information. | Page 4 |
| Study risk of bias assessment | 11 | Specify the methods used to assess risk of bias in the included studies, including details of the tool(s) used, how many reviewers assessed each study and whether they worked independently, and if applicable, details of automation tools used in the process. | Page 4 |
| Effect measures | 12 | Specify for each outcome the effect measure(s) (e.g. risk ratio, mean difference) used in the synthesis or presentation of results. | Page 4 |
| Synthesis methods | 13a | Describe any methods used to explore possible causes of heterogeneity among study results (e.g. subgroup analysis, meta-regression) | No subgroup analyses were planned or performed. |
|  | 13b | Describe any methods required to prepare the data for presentation or synthesis, such as handling of missing summary statistics, or data conversions. | Page 4 |
|  | 13c | Describe any methods used to tabulate or visually display results of individual studies and syntheses. | Page 4 |
|  | 13d | Describe any methods used to synthesize results and provide a rationale for the choice(s). If meta-analysis was performed, describe the model(s), method(s) to identify the presence and extent of statistical heterogeneity, and software package(s) used. | Page 4 |
| Reporting bias  assessment | 14 | Describe any methods used to assess risk of bias due to missing results in a synthesis (arising from reporting biases) | Page 4 |
| Certainty assessment | 15 | Describe any methods used to assess certainty (or confidence) in the body of evidence for an outcom | Page 4 |
| **RESULTS** |  |  |  |
| Study selection | 16a | Describe the results of the search and selection process, from the number of records identified in the search to the number of studies included in the review, ideally using a flow diagram. | Page 5 Figure 1 |
|  | 16b | Cite studies that might appear to meet the inclusion criteria, but which were excluded, and explain why they were excluded. | Page 5 |
| Study characteristics | 17 | Cite each included study and present its characteristics | Page 4-5, Table 1 |
| Risk of bias in studies | 18 | Present assessments of risk of bias for each included study | Supplementary Table S4 |
| Results of  individual studies | 19 | For all outcomes, present, for each study: (a) summary statistics for each group (where appropriate) and (b) an effect estimate and its precision (e.g. confidence/credible interval), ideally using structured tables or plots. | Table 2 Figures 2-5 |
| Results of  syntheses | 20a | Report the pooled effect size, confidence interval, P value, and heterogeneity for each meta-analysis. | Page 5,6 |
|  | 20b | Report the results of the subgroup and sensitivity analyses. | Not performed |
| Reporting biases | 21 | Present assessments of risk of bias due to missing results (arising from reporting biases) for each synthesis assessed. | Not assessed due to limited number of included studies (<10)” |
| Certainty of evidence | 22 | Present assessments of certainty (or confidence) in the body of evidence for each outcome assessed | Not performed |
| **DISCUSSION** |  |  |  |
| Discussion | 23a | Provide a general discussion of the main findings, taking into account the study objectives, limitations, and quality of evidence. | Page 6,7 |
|  | 23b | Discuss the similarities and differences between the results of this study and previous evidence. | Page 11 |
| Limitations | 24 | Discuss the limitations of the review itself (such as search limitations, publication bias, etc.). | Page 10,11 |
| Conclusion | 25 | Present the conclusions and discuss the implications for practice and policy. | Page 11 |
| **Other** |  |  |  |
| Registration and Plan | 26a | Provide registration information (such as the PROSPERO registration number), or indicate that the study has not been registered. | Page 3 |
|  | 26b | Indicate whether the proposal is consistent with the report, or provide a link to the proposal. | Not applicable |
| Support | 27 | Describe sources of financial or nonfinancial support for the review, and the role of the funders or sponsors in the review. | Page 12 |

Supplementary Table S3. PRISMA 2020 for Abstracts checklist (12 items)

| **Section** | **Item** | **Checklist item** | **Reported?** | **Location in abstract** |
| --- | --- | --- | --- | --- |
| Title | 1 | Identify the report as a systematic review. | Yes | Title contains "a systematic review" |
| Background | 2 | Provide an explicit statement of the main background and objectives. | Yes | Abstract, sentences 1–3 |
| Methods | 3 | Specify the inclusion criteria (PICO). | Yes | Abstract mentions "PSD patients" and "inflammatory indicators" |
| Methods | 4 | Specify the information sources (e.g., databases, registers) used and the date when the search was last performed. | Yes | Abstract mentions "PubMed, Google Scholar, Web of Science, and CNKI" and "up to January 31, 2026" |
| Methods | 5 | Specify the methods used to assess risk of bias in the included studies. | Yes | Abstract states "Quality was assessed using the Newcastle‒Ottawa Scale (NOS)" |
| Results | 6 | Specify the number of included studies and participants. | Yes | Abstract mentions "9 studies involving 2,626 patients" |
| Results | 7 | Present the main results, including the synthesized effect estimates with confidence intervals. | Yes | Abstract reports SMD and 95% CI for NLR, PLR, LMR, and SII |
| Results | 8 | Present the measures of heterogeneity (e.g., I²) for the main results. | Yes | Abstract includes I² values for each marker (e.g., I² = 49% for NLR, 90% for PLR, 63% for LMR, 70% for SII) |
| Results | 9 | Present the results of risk of bias assessment. | Yes | Abstract states "Most included studies were rated as moderate-to-high quality based on NOS scores" |
| Discussion | 10 | Provide a brief summary of the evidence and its limitations. | Yes | Abstract, final paragraph |
| Discussion | 11 | Provide a brief interpretation of the findings and their implications (applicability). | Yes | Abstract states "These findings provide a basis for early risk assessment and clinical identification of PSD" |
| Other | 12 | Provide registration information (if available), or state that the review was not registered. | Yes | Abstract states "This review was not registered in PROSPERO" |
